# Targeting the adenosinergic system in restless legs syndrome: A pilot, “proof-of-concept” placebo-controlled TMS-based protocol

**DOI:** 10.1371/journal.pone.0302829

**Published:** 2024-05-10

**Authors:** Giuseppe Lanza, Michele Salemi, Maria P. Mogavero, Valentina Catania, Annalisa Galeano, Angelo Garifoli, Bartolo Lanuzza, Manuela Morreale, Mariangela Tripodi, Mariagiovanna Cantone, Francesco Cappellani, Carmen Concerto, Alessandro Rodolico, Manuela Pennisi, Rita Bella, Raffaele Ferri

**Affiliations:** 1 Oasi Research Institute-IRCCS, Troina, Italy; 2 Department of Surgery and Medical-Surgical Specialties, University of Catania, Catania, Italy; 3 Vita-Salute San Raffaele University, Milan, Italy; 4 Division of Neuroscience, San Raffaele Scientific Institute, Sleep Disorders Center, Milan, Italy; 5 Neurology Unit, Policlinico University Hospital “G. Rodolico-San Marco”, Catania, Italy; 6 Ophtalomolgy Unit, Policlinico University Hospital “G. Rodolico-San Marco”, Catania, Italy; 7 Psychiatry Unit, Policlinico University Hospital “G. Rodolico-San Marco”, Catania, Italy; 8 Department of Biomedical and Biotechnological Sciences, University of Catania, Catania, Italy; 9 Department of Medical and Surgical Sciences and Advanced Technologies, University of Catania, Catania, Italy; University of Florida, UNITED STATES

## Abstract

Restless Legs Syndrome (RLS) is a common sleep disorder characterized by an urge to move the legs that is responsive to movement (particularly during rest), periodic leg movements during sleep, and hyperarousal. Recent evidence suggests that the involvement of the adenosine system may establish a connection between dopamine and glutamate dysfunction in RLS. Transcranial magnetic stimulation (TMS) is a non-invasive electrophysiological technique widely applied to explore brain electrophysiology and neurochemistry under different experimental conditions. In this pilot study protocol, we aim to investigate the effects of dipyridamole (a well-known enhancer of adenosinergic transmission) and caffeine (an adenosine receptor antagonist) on measures of cortical excitation and inhibition in response to TMS in patients with primary RLS. Initially, we will assess cortical excitability using both single- and paired-pulse TMS in patients with RLS. Then, based on the measures obtained, we will explore the effects of dipyridamole and caffeine, in comparison to placebo, on various TMS parameters related to cortical excitation and inhibition. Finally, we will evaluate the psycho-cognitive performance of RLS patients to screen them for cognitive impairment and/or mood-behavioral dysfunction, thus aiming to correlate psycho-cognitive findings with TMS data. Overall, this study protocol will be the first to shed lights on the neurophysiological mechanisms of RLS involving the modulation of the adenosine system, thus potentially providing a foundation for innovative “pharmaco-TMS”-based treatments. The distinctive TMS profile observed in RLS holds indeed the potential utility for both diagnosis and treatment, as well as for patient monitoring. As such, it can be considered a target for both novel pharmacological (i.e., drug) and non-pharmacological (e.g., neuromodulatory), “TMS-guided”, interventions.

## Introduction

Restless Legs Syndrome (RLS) is a common neurological disorder, affecting approximately 5% of people in the USA and Europe, who experience symptoms at least weekly [1]. Typical clinical manifestations include a periodic, rest-induced, predominantly nocturnal urge to move the legs, periodic leg movements during sleep (PLMS), and hyperarousal. Despite the absence of significant complaints of excessive daytime sleepiness, RLS patients exhibit poor total sleep time and quality, which affects also diurnal activity and functioning [2]. The current understanding of RLS pathophysiology involves two distinct yet interrelated neurotransmission systems, namely dopamine (DA) and glutamate (Glu), each primarily associated with the two main clinical features of RLS: PLMS and hyperarousal, respectively. Nevertheless, recent experimental evidence suggests that alterations in adenosine activity may provide a link between these two systems in RLS [3]. Adenosine, a key endogenous sleep-promoting factor, serves indeed as a potent modulator of both DA and Glu neurotransmission [4].

Dipyridamole, a non-selective inhibitor of nucleoside transporters ENT1 and ENT2, increases extracellular adenosine levels. In a recent open-label clinical trial, untreated primary RLS patients receiving dipyridamole demonstrated significant improvement in clinical complaints, PLMS, and total sleep time [5]. Conversely, caffeine, a well-known arousal-promoting psychostimulant, acts as a non-selective adenosine receptor antagonist. Metabolized to paraxanthine and theophylline, caffeine produces arousal effects through partial (25-50%) and non-selective blockade of adenosine receptors [6]. Recently, daily mocha coffee intake was associated with higher cognitive and mood status in non-demented patients with geriatric depression and vascular cognitive impairment, with a significant dose-response association even with moderate consumption [7,8].

In this context, genome-wide association studies have indicated an increased risk of RLS in populations with genomic variants of the protein tyrosine phosphatase D-type cell adhesion molecule receptor (PTPRD). As such, PTPRD, expressed abundantly by DA neurons, may play a crucial role in reconfiguring neural circuits, including interactions between homomeric and heteromeric G-protein-coupled receptors that mediate dopaminergic modulation [9]. Long-term activation of a DA receptor subtype may increase the expression of receptor subtypes with opposing modulatory actions, indicating the significance of dopaminergic interactions at both spinal and supraspinal levels in RLS. These interactions may extend to adenosine A1 and A2A receptors, which are prominent in the striatum, a key structure involved in RLS [10], thus offering new potential pharmacological targets for RLS treatment [9].

While promising, however, the neurophysiological correlates of the neurochemistry underlying RLS remain poorly explored. Transcranial magnetic stimulation (TMS) is a non-invasive neurophysiological technique allowing a “real-time” access to the excitatory and inhibitory electrophysiological properties of cortical and cortico-subcortical neuronal networks *in vivo* [11]. In contemporary research, various protocols are commonly employed to assess brain physiology under diverse experimental conditions, encompassing neuromodulatory interventions [12] and pharmacological manipulations [13]. Within this context, only a limited number of previous studies have indicated that global cortico-motor excitability to TMS appears unaffected by caffeine in healthy subjects [14–16]. However, two more recent papers suggest that a gamma-amino-butyric acid (GABA)-mediated index of cortical inhibition, specifically the cortical silent period (CSP) [17], and a measure of excitation mainly mediated by glutamate, i.e., the intracortical facilitation (ICF) were reduced [18]. Conversely, to the best of our knowledge, no TMS study has been conducted on dipyridamole.

Regarding the application of TMS in sleep, in recent years, several studies have investigated electrocortical patterns in response to TMS in different sleep disorders [19,20], including RLS. These studies are based on evidence demonstrating a circadian-modified excitability of the primary motor cortex (M1), sensory-motor network, and cortico-spinal tract in individuals with RLS [21,22]. Specifically, RLS exhibits a global profile of cortical hyperexcitability, which has been primarily attributed to increased motor cortex excitability and abnormal synaptic plasticity compared to healthy sleepers, although a “disinhibition” (i.e., mainly due to a reduced GABAergic tone) may contribute [22,23]. Notably, this finding appears to be specific to RLS rather than a generic reflection of sleep architecture alterations, thus allowing to draw a relatively specific RLS pattern to TMS, at least in those with a primary disorder and not affected by other sleep problems [19,24]. These findings align with electroencephalography (EEG) evidence of a hyperarousal state at the sleep onset of RLS subjects [25]. This hyperexcitability, partly resulting from electrocortical disinhibition, may also have therapeutic implications and be a potential target for neuromodulatory interventions [12]. Within this framework, a recent study has demonstrated impairment in the GABA circuitry associated with disrupted neuroplasticity in patients compared to controls [23].

Lastly, it is well-established that sleep plays a crucial role in both cognition and mood regulation. However, it remains uncertain whether cognitive impairment in some RLS patients would be solely a consequence of chronic sleep loss and fragmentation or an additional feature of the syndrome [26]. Nonetheless, there is evidence suggesting that RLS patients may exhibit cognitive deficits, particularly in memory, attention, and executive functions [27–29], also in the pediatric age [30,31]. This finding has also been linked to alterations in adenosine neurotransmission, supported by the evidence that adenosine receptors exert a significant role in modulating and adapting various homeostatic brain functions, including cognitive abilities [32,33].

Based on these considerations, a combination of dipyridamole and caffeine in the modulation of adenosine system, in both directions (i.e., up- or down-regulation, respectively), might be apply for a new and “pharmaco-TMS”-based treatment of RLS. Therefore, in this pilot study protocol, we aim to analyze the differential effects of dipyridamole and caffeine, compared to a placebo, on a number of specific measures of cortical excitability to TMS in patients with primary RLS. Initial assessments will include single- and paired-pulse TMS to evaluate cortical excitability. Then, based on the obtained measures, we will investigate the effects of dipyridamole and caffeine on TMS parameters of cortical excitation and inhibition in RLS patients. Lastly, we will assess psycho-cognitive performance in RLS patients to screen them for cognitive impairment and/or mood-behavioral dysfunction, which will eventually allow to correlate psycho-cognitive scores and TMS data.

## Materials and methods

### Study design

The study employs a double-blind, randomized, cross-over exploratory design. Each subject will receive dipyridamole, caffeine, or a placebo in a random sequence determined by computer-generated random numbers. The administration will occur during three different TMS sessions in the late afternoon (in accordance with the circadian distribution of RLS symptoms), with a sufficient wash-out period (at least 2 times the half-life of each substance) between sessions. To enhance the randomness of the sequence, planned restrictions will be unavailable to the contributors enrolling participants or assigning interventions. The allocation sequence will be implemented using sequentially numbered, opaque, sealed envelopes. Blinding of trial participants, outcome assessors, and data analysts will be maintained also after assignment to interventions.

**Fig 1:**
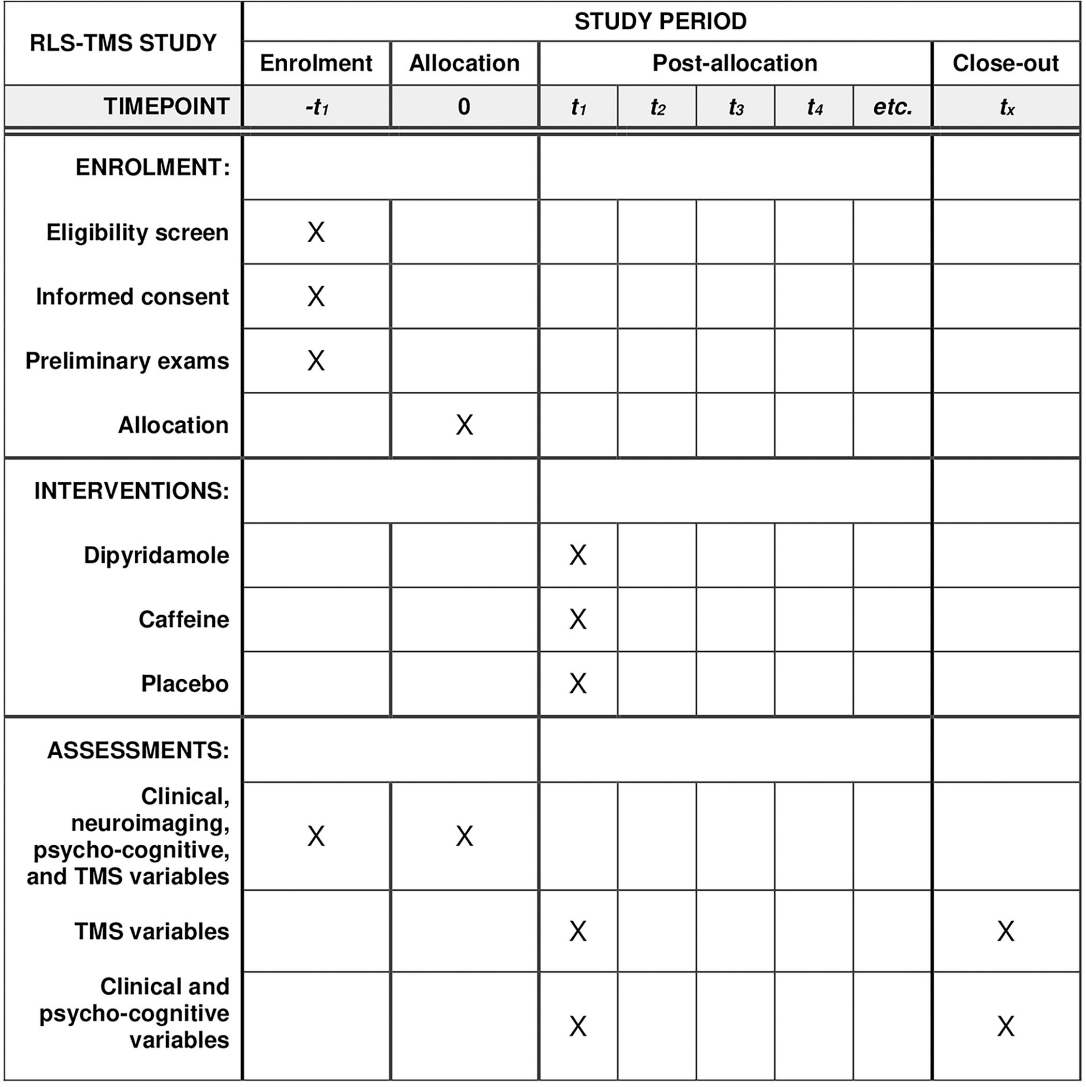
SPIRIT schedule. RLS = Restless Legs Syndrome; TMS = transcranial magnetic stimulation.

This study (version v.1.0_14/07/2020) received approval from the research Ethics Committee “CE IRCCS Sicilia – Oasi Maria SS.” on October 25, 2016 (Approval ID: 2016/CE-IRCCS-OASI/1). It adheres to SPIRIT (Standard Protocol Items for Randomized Trials) recommendations in a clinical trial protocol and related documents (Fig 1), and it has been registered in a WHO approved registry, i.e., the ISRCTN registry (registration number: ISRCTN11559397). Any protocol amendments or relevant modifications (such as changes to eligibility criteria, outcomes, analyses) will be communicated to relevant parties, including investigators, the research Ethics Committee, trial participants, trial registries, journals, and regulators. The recruitment period for this study will range from March 01, 2024, to February 28, 2025. Written informed consent will be signed by all subjects prior to the inclusion in the study; additional consent provisions for participant data collection and use will be obtained, if necessary. Authorship eligibility of protocol contributors will follow the guidelines of the International Committee of Medical Journal Editors (ICMJE).

### Participants

Patients will be recruited from a community clinic at the Department of Neurology IC and Sleep Research Center of the Oasi Research Institute-IRCCS in Troina (Italy), upon their first diagnosis. They must meet all the inclusion criteria and none of the exclusion criteria, whether as out-patients or in-patients. Recruitment and clinical assessment will be supervised by a trained neurologist expert in sleep medicine and disorders (R.F.). Given the novelty of the study, sample size calculation relies on similar articles in the literature and the typical sample size in TMS research, indicating a minimum of 30 *de novo*, drug-free, patients. All strategies for achieving participant enrolment to reach target sample size will be adopted, as well as plans to promote participant retention and follow-up completion.

Inclusion criteria will be: i) age 18-65 years; ii) diagnosis of RLS according to the latest criteria by the International RLS Study Group [34]; iii) RLS symptoms on 3 or more days per week for at least 3 months; iv) International RLS Rating Scale (IRLS-RS) score >15 (i.e., moderate symptomatology, at least); v) normal brain magnetic resonance imaging (MRI) and upper limb electromyography (EMG), including the study of the F-waves; vi) women of childbearing potential must have a negative pregnancy test and agree not to become pregnant during the whole experimental procedure; vii) personally signed and dated informed consent obtained before any procedure. All personal information about potential and enrolled participants will be collected and maintained, in order to protect confidentiality before, during, and after the whole procedure.

Exclusion criteria will be: i) drug intake affecting cortical excitability or cognitive performance, including those used for RLS; ii) Mini Mental State Examination score <24; iii) any major psychiatric disease; iv) other neurological or sleep disorders; v) severe, untreated, acute, or not compensated medical illness; vi) any secondary form of RLS (i.e. renal failure, anemia, low serum iron and ferritin levels, pregnancy, peripheral neuropathy, etc.); vii) contraindication to dipyridamole and/or caffeine intake; viii) any condition precluding MRI or TMS execution, such as the presence of a cardiac pacemaker, defibrillator, or mechanical valve, presence of non-compatible joint prostheses, previous neurosurgical procedures, and, for TMS only, seizure or history of epilepsy [35]. A conventional EEG will be also performed to exclude a predisposition to seizure.

### Assessment

This is a single center, double blind, randomized, 3-period 3-treatment, random-sequence crossover study. After the baseline TMS assessment, the same participants will be orally exposed to all substances (dipyridamole, caffeine, or placebo), in a random order, after a washout period. Participants and researchers involved will be blind to the substance’s nature. More in details, the “dipyridamole arm” will consist of a single oral dose of dipyridamole 75 mg, while the “caffeine arm” of a single oral dose of caffeine 200 mg. After an approximately one-hour interval (the absorption peak of dipyridamole and caffeine is known to occur in about 60 minutes after the oral intake), TMS tests will be repeated to evaluate potential effects of the active compounds compared to placebo. All capsules (active and placebo), prepared by the Pharmacy Department of the Oasi Research Institute-IRCCS, will be indistinguishable. Clinical evaluation and IRLS-RS will be repeated after each experimental condition. Discontinuation or modification of interventions will be allowed, if necessary (e.g., dose change in response to harms or worsening disease), and all the procedures needed to improve adherence to intervention protocols and monitoring will be taken into account. There will be no other relevant or concomitant treatment during the interventions.

Cognitive assessment, will be performed in the morning by a trained psychologist expert in clinical neuropsychology (V.C.). Tests will cover a range of different cognitive domains, including attention, memory, verbal fluency, executive functions, and working memory. In addition, all subjects will complete: the vocabulary subtest of the Wechsler Adult Intelligence Scale, which assesses the patients’ understanding of words and reflects language development, expressive language skills, cultural and educational experiences, ability to use words appropriately, and retrieval of information from long-term memory; the Global Sleep Assessment Questionnaire, which distinguishes between sleep disorders (including no sleep disorder), also serving as a practical screening tool for primary care and sleep centers; the Pittsburgh Sleep Quality Index, an internationally validated rating scale used to provide a reliable, valid, and standardized measure of sleep quality; the Beck Depression Inventory II, a multiple-choice self-report inventory, which is one of the most widely used psychometric tests for measuring the severity of depression; the State-Trait Anxiety Inventory, which is a self-report rating scale that assesses separate dimensions of “state” and “trait” anxiety (examples of what this inventory measures include feelings of apprehension, tension, nervousness, and worry). Overall, this comprehensive battery will be used in order to assess the overall premorbid intelligence and the quality of life (QoL) of all participants, as well as to screen them for any relevant co-morbid psychiatric condition. Psycho-cognitive tests will not be repeated because of the very short time interval (one hour) compared to the first assessment.

Single-pulse TMS will be performed by using a High-power Magstim 200 mono-pulse magnetic stimulator (The Magstim Company, Whitland, Dyfed, UK). The stimulator will be connected to a 70 mm figure-of-eight coil, held over the M1 at the optimum scalp position to elicit motor evoked potentials (MEPs) in the contralateral First Dorsal Interosseous (FDI) muscle of the dominant hand, according to the Edinburgh Handedness Inventory [36]. Paired-pulse TMS will be run by connecting two magnetic stimulators to each other through a “BiStim” module (The Magstim Company, Whitland, Dyfed, UK). EMG activity will be recorded from silver/silver chloride surface electrodes. Motor responses will be amplified and filtered (bandwidth 3-3,000 Hz) using a 2-channel amplifier system, with an amplification factor of the screen of 100 μV per division unit for the measurement of rMT, and 1 mV per division unit during MEP recording. The temporal resolution of the screen will be 5 ms per division unit, in such a way that TMS artifact, the beginning, and the end of MEP are always visible.

Single-pulse TMS measures will include: resting motor threshold (rMT), CSP, MEP latency and amplitude, and central motor conduction time (CMCT). Resting MT is defined, according to the international recommendations [37], as the lowest stimulus intensity able to elicit MEP of an amplitude >50 μV in at least 5 of 10 trials, with the muscle at rest. This is a global measure of excitability, reflecting the membrane ion channel functioning and the Glu-mediated excitation state of cortico-spinal neurons and interneurons projecting onto these neurons within the M1, as well as the excitability of spinal motor neurons, neuromuscular junctions, and muscles [38]. CSP is determined as approximately 50% of maximum tonic voluntary contraction of the target muscle, induced by contralateral TMS delivered at 130% of rMT. During CSP recording, the subject will maintain the isometric tonic contraction by abducting the index finger against a strain gauge. The mean CSP duration, based on trial-by-trial measurements of 10 rectified traces, will be calculated. Following the above-mentioned guidelines [37], in each single trial, CSP is measured as the time elapsing from the onset of MEP until the recurrence of voluntary tonic EMG activity. CSP is primarily mediated by the activity of GABA-B intracortical inhibitory interneurons [39]. MEPs with the shortest latency (defined as the temporal interval between TMS artifact and the first negative deflection of the evoked motor response) will be considered for analysis, given that it is known to best reflect the conductivity along the fastest cortico-spinal axons. MEPs size is calculated as the biggest peak-to-peak amplitude of the motor responses [37]. CMCT reflects the integrity of the cortico-spinal conductivity, from the upper to the lower motor neurons. It is calculated by subtracting the conduction time in peripheral nerves from MEP latency obtained during moderate active muscle contraction (10-20% of maximum background force), at a stimulus intensity set at 130% of rMT [37].

Intracortical circuits will be studied by using the conventional conditioning-test paradigm by Kujirai and co-workers [40]. The procedure consists of applying two magnetic stimuli in rapid succession through two magnetic stimulators connected to each other. The conditioning stimulus (subthreshold) is applied at 80% of the subject’s rMT, and the intensity of the test stimulus (suprathreshold) is set at 130% of rMT. The interstimuls intervals (ISIs) tested will be: 2, 3, 5, 10, 15, and 20 ms; ten trials for each ISI will be recorded randomly. The responses are expressed as the ratio of MEP amplitude produced by paired stimulation to that produced by test stimulation alone. Short-latency intracortical inhibition (SICI) is obtained at short ISIs (2, 3, and 5 ms) in which the conditioning stimulus determines a motor suppression with respect to the test stimulus; it is attributed to an activation of the GABA-A neuronal transmission [41]. ICF is studied at longer ISIs (10, 15, and 20 ms), in which the conditioning stimulus determines an enhancement of the motor response with respect to the test stimulus. ICF is a more complex phenomenon, modulated by multiple neurotransmitters, although it mainly involves the excitatory Glu neurons, with a DAergic contribution 04 [42].

All TMS measurements will be conducted by a trained operator, expert in non-invasive brain stimulation techniques (G.L.), while subjects are seated in a comfortable chair with continuous visual and EMG monitoring to ensure either a constant level of muscular activity during tonic contraction or complete relaxation at rest. Data will be collected on a dedicated database and stored for the off-line analysis [43]. Data entry, coding, security, and storage, including processes to promote data quality (such as double data check and range checks for data values) will be carefully considered. Data could not be fully anonymized, although they will be pseudonymized and safely stored. Similarly, any adverse effect during or after the procedure will be carefully collected, assessed, reported, and managed, if necessary.

When appropriate, patients will be actively involved in the implementation and refinement of the study protocol, e.g., by providing any input on the various parts of the experimental procedure and customizing the relevant outcomes. Although limitations exist due to data protection and technical requirements, efforts will be made to involve patients also in the development of the dissemination strategy, including writing a plain language summary and designing a leaflet for peers and patient groups/associations. Trial results will be communicated to participants, healthcare professionals, and relevant groups through scientific publications, conferences, seminars, and other data-sharing arrangements.

### Outcomes

Primary outcomes: rMT (%), CSP (ms), MEP latency (ms) and amplitude (mV), CMCT (ms), SICI (ratio), and ICF (ratio). Secondary outcomes: correlation between TMS metrics (primary outcomes) and psycho-cognitive measures (Vocabulary subtest of the Wechsler Adult Intelligence Scale; Global Sleep Assessment Questionnaire; Pittsburgh Sleep Quality Index; Beck Depression Inventory II; State-Trait Anxiety Inventory).

### Statistical analysis

After the analysis of descriptive statistics, based on the results of the Shapiro–Wilk normality test, each outcome will be compared across the three treatment conditions by a repeated-measures ANOVA (if data are normally distributed) or the nonparametric Friedman ANOVA (if data are not distributed normally). After the completion of the data collection, a power analysis will be carried out in order to check if the statistical power of at least 80% is achieved and plan an eventual study extension is needed or not. In any case, the planned number of patients will allow to obtain a power of 80% (with alpha 0.05) even with a “moderate” effect size, as small as 0.465 [44]. No missing data are expected because we plan to continue the recruitment until we reach a number of 30 participants with a complete research protocol.

**Fig 2:**
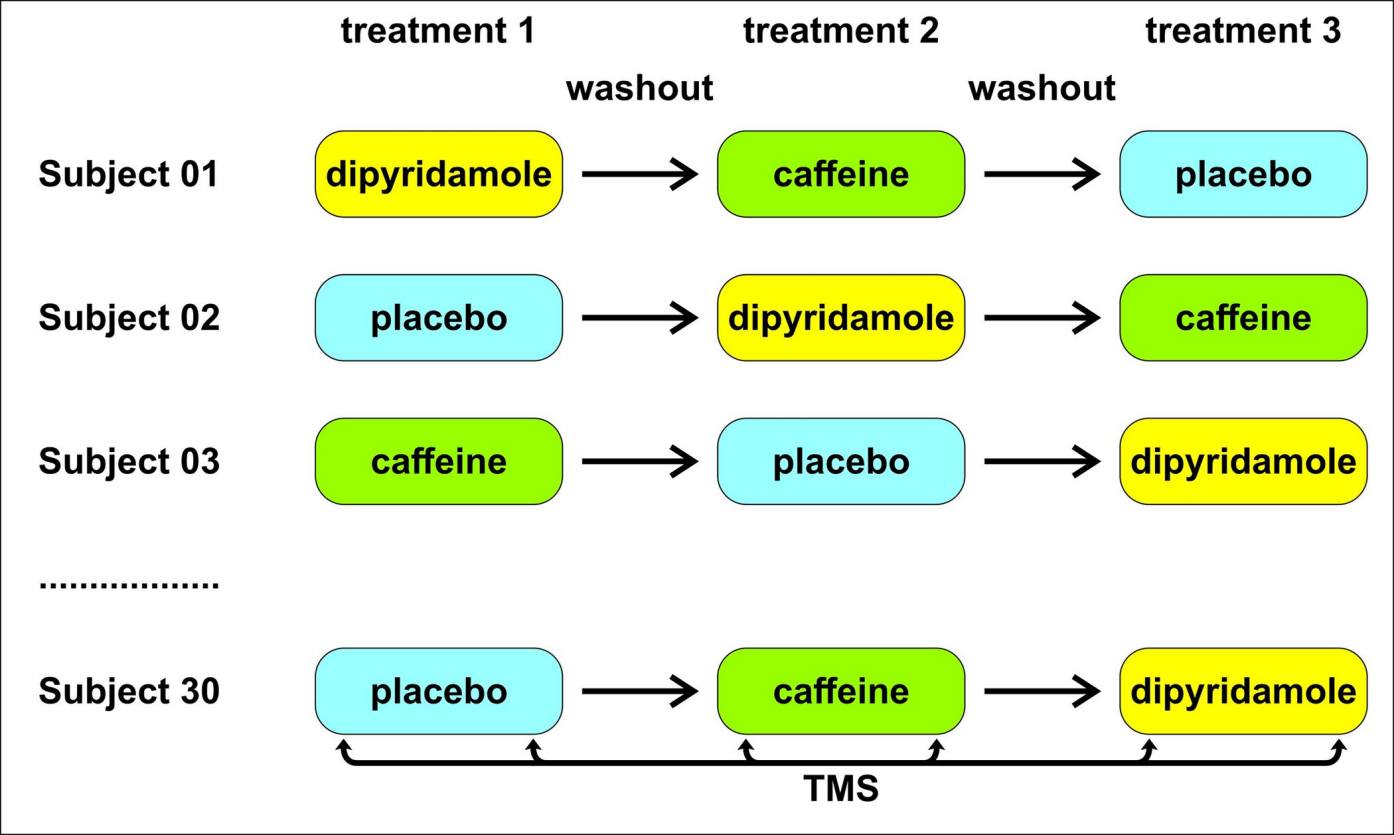
Flow chart and main procedures of the study protocol. TMS = transcranial magnetic stimulation.

Fig 2 illustrates the flow chart and main procedures of the study protocol.

## Expected results

This study protocol will be the first to shed lights on the neurophysiological mechanisms of RLS involving the modulation of the adenosine system, thus potentially providing a foundation for innovative “pharmaco-TMS”-based treatments. The distinctive TMS profile observed in RLS holds indeed the potential utility for both diagnosis and treatment, as well as for patient monitoring. As such, it can be considered a target for both novel pharmacological (i.e., drug) and non-pharmacological (e.g., neuromodulatory), “TMS-guided”, interventions [45].

Dipyridamole-induced activation of the adenosinergic system is postulated to effectively modulate cortico-spinal excitability and plasticity in RLS, offering a potential expansion of therapeutic options for this sleep disorder [46]. The integration of TMS with clinical, cognitive, sleep-related, and advanced neuroimaging data might serve as a noninvasive neurophysiological tool for studying RLS and designing innovative drugs applicable in daily clinical practice. Furthermore, the study’s exploration of adenosine in sleep and cognition provides a rationale for investigating cognitive aspects of RLS and translating TMS measures to patients at risk for cognitive impairment.

The study’s findings are expected to support recent evidence suggesting that RLS mechanisms involve not only the hypothalamus-spinal dopaminergic circuit, but also basal ganglia circuits and limbic system areas. Structural alterations in RLS may extend to the morphology and volume of these structures [10], emphasizing the need for a comprehensive reconsideration of the complex neurophysiological and neurochemical mechanisms underlying RLS. As such, the identification of new neurophysiological targets, such as modulating the adenosinergic system, presents an exciting avenue for innovative treatments applicable in daily clinical practice.

Building on recent preclinical and clinical studies, we anticipate reinforcing the role of a diminished adenosinergic tone in the pathomechanism of RLS, extending this understanding to the neurophysiological level. This exploration may pave the way for future, more targeted, and “TMS-guided” therapeutic interventions. Additionally, beyond adenosine, various neurotransmitters implicated in RLS, such as DA, Glu, GABA, acetylcholine, and opioids, can be examined through TMS. These neurotransmitters may exert modulatory effects within the cortical sensory-motor network and the cortico-spinal tract.

Potential limitations of this study protocol include:

1. TMS may not provide disease-specific pathophysiological information, although it is sensitive to various neurotransmitters and cortical-subcortical motor inputs; therefore, instead of seeking a single marker of the disease process, the study will aim to identify a distinctive panel of measures [47], potentially allowing the identification of neurophysiological indexes related to adenosine responders;
2. the study will be conducted in the awake state, differing from the sleep condition; however, as previously reviewed [19,21,22], most studies, even the most recent ones, are performed during wakefulness, likely due to technical/procedural reasons;
3. the use of a hand muscle in RLS subjects might be considered inadequate, although TMS measures have shown involvement even when recording over hand muscles [19,21]; moreover, technical difficulties of MEPs recordings from lower limbs may affect the reliability and reproducibility of results;
4. the minimum number of participants cannot be determined through statistical sample size/power analysis due to the novelty of the proposal and the lack of previous data, although comparable numbers of participants are common in TMS literature; nevertheless, if preliminary data analysis will suggest the need to increase the total sample size beyond 30 after recruiting the first 10-15 patients, it will be done without further funding requests;
5. the possibility of a “placebo effect” cannot be entirely excluded, but it is expected to do not to persist over time.

## Conclusions

Through a customized “pharmaco-TMS” design, this pilot study protocol aims to provide clinically, psycho-cognitively, and electrophysiologically relevant findings with translational value for implementing pharmacological management in RLS patients [3,5]. Given the significant epidemiological impact of RLS, further studies and longitudinal investigations are warranted to establish a potential causal relationship between dipyridamole/caffeine-induced changes and the sleep/QoL of RLS patients. In this scenario, TMS is a feasible neurophysiological translational tool capable of exploring the pathophysiology and neurochemistry of RLS and other sleep disorders, non-invasively probing cortico-spinal excitability, functional connectivity, and synaptic plasticity in the human brain [48].

## Supporting information

S1 Checklist SPIRIT 2013 checklist: Recommended items to address in a clinical trial protocol and related documents*. (DOC)

S1 File (PDF)
