# Supplementary material for: Targeting the adenosinergic system in restless legs syndrome: A pilot, “proof-of-concept” placebo-controlled TMS-based protocol
Source: PLoS One. 2024 May 10;19(5):e0302829. doi: 10.1371/journal.pone.0302829 (PMC11086884; doi:10.1371/journal.pone.0302829)
Supplement: S1 File — (PDF) [file pone.0302829.s002.pdf]

ISRCTN11559397 https://doi.org/10.1186/ISRCTN11559397

Neuromodulation of sleep disorders: a new neurophysiological-based approach to restless legs syndrome

Submission date	Recruitment status	✓ Prospectively registered
08/02/2024	Recruiting	? Protocol not yet added
Registration date	Overall study status	? SAP not yet added
13/02/2024	Ongoing	– Results not yet expected
Last Edited	Condition category	– Raw data not yet expected
13/02/2024	Nervous System Diseases	✓ Record updated in last year

Plain English Summary
Background and study aims
Restless Legs Syndrome (RLS) is a common sleep disorder characterized by an urge to move the legs that is responsive to movement (particularly during rest), periodic leg movements during sleep, and disrupted sleep. RLS is rather complex and not fully understood yet. Although the dopaminergic pathways play a crucial role, other neurotransmitters (brain chemicals) seem to contribute, such as the adenosinergic system. However, there isn't enough evidence yet of direct involvement in RLS patients. Transcranial magnetic stimulation (TMS) is a non-invasive method used to study brain activity and chemistry in various neurological conditions, including sleep disorders. This study aims to see how dipyridamole, which enhances adenosinergic transmission, and caffeine, which blocks adenosine receptors, affect TMS measurements of brain activity.

Who can participate?
Adult patients aged 18-65 years with primary RLS

What does the study involve?
Each participant will receive a single oral dose of dipyridamole, caffeine, or a placebo in a random sequence. Drug administration will occur during three different TMS sessions in the late afternoon with a sufficient wash-out period between sessions.

What are the possible benefits and risks of participating?
Although the study has mainly a diagnostic purpose, the results will also support the design of new evidence-based drugs and other treatment approaches.

Where is the study run from?
Oasi Research Institute-IRCCS (Italy)

When is the study starting and how long is it expected to run for?
March 2024 to February 2025

Who is funding the study?
Ministry of Health (Italy)

Who is the main contact?
Dr Giuseppe Lanza, giuseppe.lanza1@unict.it

Contact information

Type(s)
Scientific, Principal Investigator

Contact name
Prof Giuseppe Lanza

ORCID ID
http://orcid.org/0000-0002-5659-662X

Contact details
Via Conte Ruggero, 73
Troina
Italy
94018
+39 (0)935 936930
glanza@oasi.en.it

Type(s)
Public

Contact name
Dr Rosa Di Giorgio

ORCID ID
http://orcid.org/0000-0002-5659-662X

Contact details
Via Conte Ruggero, 73
Troina
Italy
94018
+39 (0)935 936374
dir.scien@oasi.en.it

Additional identifiers

EudraCT/CTIS number
Nil known

IRAS number

ClinicalTrials.gov number

Nil known

Protocol/serial number
Nil known

Study information

Scientific Title
Targeting the adenosinergic system in restless legs syndrome: a “proof-of-concept” placebo-controlled transcranial magnetic stimulation-based protocol

Acronym
RLS-TMS

Study hypothesis
In this study protocol, the aim is to investigate the effects of dipyridamole (a well-known enhancer of adenosinergic transmission) and caffeine (an adenosine receptor antagonist) on measures of cortical excitation and inhibition in response to transcranial magnetic stimulation (TMS) in patients with primary restless legs syndrome (RLS). Initially, the researchers will assess cortical excitability using both single- and paired-pulse TMS in patients with RLS. Then, based on the measures obtained, they will explore the effects of dipyridamole and caffeine, in comparison to placebo, on various TMS parameters related to cortical excitation and inhibition. Finally, the researchers will evaluate the psycho-cognitive performance of RLS patients to screen them for cognitive impairment and/or mood-behavioral dysfunction, thus aiming to correlate psycho-cognitive findings with TMS data.

Ethics approval required
Ethics approval required

Ethics approval(s)
Approved 25/10/2016, Comitato Etico dell'IRCCS Associazione Oasi Maria SS (Via Conte Ruggero, 73, Troina, 94018, Italy; +39 (0)935 936374; dir.scien@oasi.en.it), ref: 2016/CE-IRCCS-OASI/1

Study design
Double-blind randomized cross-over exploratory design

Primary study design
Interventional

Secondary study design
Randomised cross over trial

Study setting(s)
Hospital, Medical and other records

Study type(s)
Diagnostic, Efficacy

Participant information sheet

Not available in web format, please use contact details to request a participant information sheet

Condition
Non-invasive exploration of the pathophysiology and neurochemistry of restless legs syndrome

Interventions
Each participant will receive dipyridamole, caffeine, or a placebo in a random sequence determined by computer-generated random numbers. The administration will occur during three different TMS sessions in the late afternoon (in accordance with the circadian distribution of RLS symptoms), with a sufficient wash-out period (at least 2 times the half-life of each substance) between sessions. To enhance the randomness of the sequence, planned restrictions will be unavailable to the contributors enrolling participants or assigning interventions. The allocation sequence will be implemented using sequentially numbered, opaque, sealed envelopes. Blinding of trial participants, outcome assessors, and data analysts will be maintained also after assignment to interventions.

Dipyridamole arm: dipyridamole; dosage given: 75 mg; method and frequency of administration: single oral administration; total duration of treatment: 1 day; follow-up: none.

Caffeine arm: caffeine; dosage given: 200 mg; method and frequency of administration: single oral administration; total duration of treatment: 1 day; follow-up: none.

Intervention Type
Mixed

Primary outcome measure
1. Resting motor threshold (%)
2. Cortical silent period (ms)
3. Latency and amplitude of the motor evoked potentials (ms/mV)
4. Central motor conduction time (ms)
5. Intracortical inhibition (ratio)
6. Intracortical facilitation (ratio)

All these measures, elicited through specific transcranial magnetic stimulation (TMS) protocols, will be performed at baseline and 1 hour after the administration of dipyridamole, caffeine, or placebo.

Secondary outcome measures
Correlation between TMS metrics (please see those listed among primary outcome measures) and psycho-cognitive assessment, in order to screen RLS patients for cognitive impairment and /or mood-behavioral dysfunction:
1. Vocabulary assessed using the Wechsler Adult Intelligence Scale
2. Presence of sleep disorders assessed using the Global Sleep Assessment Questionnaire
3. Sleep quality assessed using the Pittsburgh Sleep Quality Index
4. Severity of depression assessed using the Beck Depression Inventory II
5. State and trait anxiety assessed using the State-Trait Anxiety Inventory

Correlations will be performed at baseline only, since psychocognitive tests are not repeated after 1 hour.

Overall study start date
01/03/2024

Overall study end date
28/02/2025

Eligibility

Participant inclusion criteria
1. Age 18-65 years
2. Diagnosis of RLS according to the latest criteria by the International RLS Study Group
3. RLS symptoms on 3 or more days per week for at least 3 months
4. International RLS Rating Scale (IRLS-RS) score >15 (i.e., moderate symptomatology, at least)
5. Normal brain magnetic resonance imaging (MRI) and upper limb electromyography (EMG), including the study of the F-waves
6. Women of childbearing potential must have a negative pregnancy test and agree not to become pregnant during the whole experimental procedure
7. Personally signed and dated informed consent obtained before any procedure. All personal information about potential and enrolled participants will be collected and maintained, in order to protect confidentiality before, during, and after the whole procedure

Participant type(s)
Patient

Age group
Adult

Lower age limit
18 Years

Upper age limit
65 Years

Sex
Both

Target number of participants
30

Participant exclusion criteria
1. Drug intake affecting cortical excitability or cognitive performance, including those used for RLS
2. Mini Mental State Examination score <24
3. Any major psychiatric disease
4. Other neurological or sleep disorders
5. Severe, untreated, acute, or not compensated medical illness
6. Any secondary form of RLS (i.e. renal failure, anemia, low serum iron and ferritin levels, pregnancy, peripheral neuropathy, etc.)
7. Contraindication to dipyridamole and/or caffeine intake
8. Any condition precluding MRI or TMS execution, such as the presence of a cardiac pacemaker, defibrillator, or mechanical valve, presence of non-compatible joint prostheses, previous neurosurgical procedures, and, for TMS only, seizure or history of epilepsy. A conventional EEG will be also performed in order to exclude a predisposition to seizure.

Recruitment start date
01/03/2024

Recruitment end date
28/02/2025

Locations

Countries of recruitment
Italy

Study participating centre
Oasi Research Institute-IRCCS
Via Conte Ruggero, 73
Troina
Italy
94018

Sponsor information

Organisation
Oasi Research Institute-IRCCS

Sponsor details
Via Conte Ruggero, 73
Troina
Italy
94018
+39 (0)935 936374
dir.scien@oasi.en.it

Sponsor type
Research organisation

Website
https://engirccs.oasi.en.it/

Funder(s)

Funder type
Government

Funder Name
Ministero della Salute

Alternative Name(s)
Italian Ministry of Health, Italy Ministry of Health, Ministry of Health of Italy, Ministry of Health - Italy, Ministry of Health, Italy

Funding Body Type
Government organisation

Funding Body Subtype
National government

Location
Italy

Results and Publications

Publication and dissemination plan
Planned publication in a high-impact peer-reviewed journal.

Intention to publish date
30/06/2025

Individual participant data (IPD) sharing plan
The datasets generated and/or analysed during the current study will be available upon request. This study (version v.1.0_14/07/2020) received approval from the Research Ethics Committee "CE IRCCS Sicilia – Oasi Maria SS." on October 25, 2016 (Approval ID: 2016/CE-IRCCS-OASI/1). It adheres to SPIRIT (Standard Protocol Items for Randomized Trials) recommendations in a clinical trial protocol and related documents. Any protocol amendments or relevant modifications (such as changes to eligibility criteria, outcomes, and analyses) will be communicated to relevant parties, including investigators, the Research Ethics Committee, trial participants, trial registries, journals, and regulators. The recruitment period for this study will range from March 01, 2024, to February 28, 2025. Written informed consent will be signed by all subjects prior to their inclusion in the study; additional consent provisions for participant data collection and use will be obtained, if necessary. Authorship eligibility of protocol contributors will follow the guidelines of the International Committee of Medical Journal Editors (ICMJE).

The name and e-mail address of the investigator/body who should be contacted for access to the datasets: "Scientific Secretary of the Oasi Research Institute-IRCCS, Troina (Italy); e-email address: dir.scien@oasi.en.it". The type of data that will be shared: "Clinical-demographic, psycho-cognitive, and TMS data". Dates of availability: "Currently not known, presumably after June 2025". Whether consent from participants was required and obtained: "Written informed consent will be signed by all subjects prior to the inclusion in the study; additional consent provisions for participant data collection and use will be obtained, if necessary.". Comments on data anonymization: "Data could not be fully anonymized, although they will be pseudonymized and safely stored".

IPD sharing plan summary

Available on request
